# Non-Communicable Diseases in Children: Systems-Based Approaches to Incorporating Nutrition into Medical Care

**DOI:** 10.3390/children12111503

**Published:** 2025-11-06

**Authors:** Michelle Walters, Ronald Barr, Joao Breda, Francesca Celletti, João de Bragança, Inge Huybrechts, Oria James, Zisis Kozlakidis, Paul Marsden, Stephen Ogweno, Roberta Ortiz, Maja Beck Popovic, Johanna Ralston, Mireya Vilar-Compte, Elena J Ladas

**Affiliations:** 1 Division of Hematology/Oncology/Stem Cell Transplantation, Department of Pediatrics, Columbia University Irving Medical Center, New York, NY 10032, USA; mw3328@cumc.columbia.edu; 2 Departments of Pediatrics, Pathology and Medicine, McMaster University, Hamilton, ON L8S 4K1, Canada; rbarr@mcmaster.ca; 3 Division of Country Health Policies and Systems, World Health Organization Regional Office for Europe, 10675 Athens, Greece; rodriguesdasilvabred@who.int; 4 Office of the Director, Nutrition and Food Safety, World Health Organization, 1211 Geneva, Switzerland; cellettif@who.int; 5 Childhood Cancer International, 1018 RG Amsterdam, The Netherlands; jb@acreditar.pt; 6 International Agency for Research on Cancer, World Health Organization, 69366 Lyon CEDEX 07, France; huybrechtsi@iarc.who.int (I.H.); kozlakidisz@iarc.who.int (Z.K.); 7 Global Diabetes Compact, World Health Organization, 1211 Geneva, Switzerland; ojames@who.int; 8 Health Workforce Department, World Health Organization, 1211 Geneva, Switzerland; marsdenp@who.int; 9 Stowelink Foundation, Nairobi 00100, Kenya; founder@stowelink.com; 10 Department of Noncommunicable Diseases, Rehabilitation and Disability, World Health Organization, 1211 Geneva, Switzerland; ortizr@who.int; 11 International Society of Paediatric Oncology, 6344 Meierskappel, Switzerland; maja.beck-popovic@chuv.ch; 12 World Obesity Federation, London EC1N 2SW, UK; jralston@worldobesity.org; 13 Health, Nutrition & Population Global Practice, World Bank, Washington, DC 20433, USA; mvilar@worldbank.org

**Keywords:** non-communicable diseases, nutrition, health systems, nutrition care, pediatrics

## Abstract

**Highlights:**

**What are the main findings?**

- Non-communicable diseases (NCDs) impact 2.1 billion children worldwide and cause nearly 1 million deaths annually, with poor nutritional status further in-creasing mortality and treatment-related complications.
- Integrating nutrition care into childhood NCD management improves survival, reduces treatment-related complications, influences disease progression, and lowers healthcare costs.

**What is the implication of the main finding?**

- Nutrition services should be embedded as a standard component of NCD care for children, yet underfunding, workforce shortages, inadequate training, and limited access to nutrition products continue to hinder progress.
- Multi-sectoral collaboration is essential to expand the nutrition workforce, strengthen training and professional standards, establish effective monitoring frameworks, and leverage technology to deliver sustainable solutions.

**Abstract:**

Non-communicable diseases (NCDs) affect over 2.1 billion children globally, accounting for 15.9% of deaths in children under 20 and contributing 174 million years lived with disability. Integrating nutrition care into NCD management within health systems can save lives, reduce costs, and improve quality of life. Nutrition interventions have been found to improve survival rates in children with cancer by 30%. Incorporating early nutrition interventions in hospitals is associated with a 36% reduction in per-patient costs. Despite these clear benefits, nutrition care is often not readily accessible as part of NCD management in children. Access to trained nutrition professionals is limited, and nutrition training for healthcare workers is often inadequate. There are cost-effective and scalable models for delivering high-quality nutrition care, but scaling these models will require commitment to capacity building, training, technological innovation, and monitoring frameworks. Coordinated, multisectoral responses are needed urgently to incorporate nutrition sustainably into healthcare systems to confront the growing burden of childhood NCDs.

## 1. Introduction

Non-communicable diseases (NCDs) affect over 2.1 billion children worldwide and account for 15.9% of deaths in children under the age of 20, resulting in approximately 1 million childhood deaths annually [1]. NCDs are responsible for 38.8% of deaths among adolescents aged 10–24 years [2] and contribute to 174 million years lived with disability in children [3]. These figures highlight the significant burden of NCDs in childhood and adolescence, and the urgent need for systematic change. An effective but often underutilized approach is integrating nutrition care into NCD management within health systems. This strategy has proven to save lives [4], lower costs [5,6,7], and lessen the burden of NCDs [8]. In recognition of this need, the International Agency for Research on Cancer, World Health Organization, the International Initiative for Pediatrics and Nutrition (IIPAN), Columbia University Irving Medical Center, and the International Society of Paediatric Oncology hosted a side event during the 78th World Health Assembly (19–27 May 2025), titled Addressing Pediatric Non-Communicable Diseases: Building Systems-Based Approaches in Nutrition, which highlighted the importance of building resilient health systems capable of delivering high-quality nutrition care to children with NCDs.

The recent high-level meeting at the United Nations reaffirmed NCDs as a high priority on the global health agenda. Yet, significant gaps remain, especially in clinician education and patient access to nutrition clinical services. Implementing food and nutrition policies remains a persistent challenge across countries. One major impediment to successful implementation is the high reliance on non-health sectors, including education, trade, finance, and commerce, rather than health systems, to deliver nutrition-related policies and interventions [9]. Programs that have sought to integrate nutrition into health systems cite barriers such as a lack of health financing for nutrition, exclusion of nutrition indicators from health management systems, and limited availability of essential nutrition supplies [10].

Children in the lowest socioeconomic strata are particularly affected as they often fully rely on public health systems [11]. To close this gap in care, incorporating nutrition services into universal health coverage is a necessary next step [12]. Achieving this integration will require sustained investment in financing mechanisms, policy frameworks emphasizing nutrition, a well-trained workforce in clinical nutrition, health information systems inclusive of nutrition indicators, and robust monitoring frameworks [12,13]. Successful examples already exist. In Rwanda, a community-based health insurance program that included nutrition services was associated with a significant reduction in stunting [14].

While nutrition is a critical determinant of health outcomes, it must be complemented by other evidence-based approaches to address childhood NCDs effectively. Interventions promoting physical activity [15] and community- and school-based health initiatives [16,17] have shown success in improving outcomes for children with NCDs. For instance, physical activity interventions among children with type 1 diabetes have been shown to improve glycemic control and insulin sensitivity [15]. Similarly, school-based interventions targeting dietary behavior and physical activity have been effective in reducing the prevalence of obesity [16]. Together, these complementary strategies highlight the benefit of multisectoral frameworks to optimize outcomes for children and adolescents with NCDs.

## 2. The Importance of Nutrition in Health Systems

The clinical impact of poor nutritional status on morbidities and mortality in children with NCDs cannot be overlooked. Up to 45% of children with chronic kidney disease experience malnutrition [18]. Among children with cancer, the prevalence of malnutrition reaches as high as 88% in Africa, 66% in Asia, and 79% in Latin America [19]. In India, over 50% of children with congenital heart disease were found to have moderate acute malnutrition [20]. Children with end-stage kidney disease, for example, have a staggering 30 times higher mortality rate compared to their healthy peers, which is exacerbated by poor nutritional status [21]. Across multiple childhood NCDs, poor nutritional status further increases the risk of mortality [22,23], infections [23], treatment-related complications [23,24], hospitalizations [22], and reduced quality of life [25,26]. In children undergoing treatment for cancer, those with malnutrition experienced up to a 2.3-fold lower survival rate compared with healthy weight peers [23]. Children with diabetic ketoacidosis and malnutrition were 4.7 times more likely to experience life-threatening hypoglycemia compared to their well-nourished peers [24]. However, evidence indicates that prevention and targeted management of nutrition risk factors can reverse or significantly reduce the adverse effects of poor nutrition on clinical outcomes across several NCDs. For children with cancer, proactive nutrition care improved survival by 30% [27]. Providing early enteral nutrition to children undergoing surgery for congenital heart disease reduced postoperative mortality by 58% [28]. Nutrition counseling resulted in a fourfold reduction in the likelihood of progressing from prediabetes to type 2 diabetes [29] and has led to measurable reductions in cholesterol levels [30]. Among children with overweight or obesity and asthma, a multidisciplinary intervention that included nutrition counseling resulted in a clinically meaningful 10% improvement in lung function [31]. Together, these data underscore the critical role of nutrition throughout the continuum of care and strengthen the case for greater investment in nutrition services within healthcare systems.

Prioritizing nutrition care also has economic benefits. However, nutrition services receive just 3% of government health budgets and provision of services remains heavily dependent on philanthropic or external funding [32], which limits long-term sustainability, as external funding streams are often short-lived [10]. In countries with national nutrition policies, a mere 39% have a costed operational plan for how to implement and finance nutrition-specific activities [12], limiting their ability to translate policies into practice. By contrast, integrating nutrition into healthcare systems can drive substantial cost savings for governments. For example, early nutrition interventions in hospitals have been linked to a 36% reduction in per-patient costs [5], with estimated national savings of US$862.6 million in healthcare expenditures [5] and US$8.1 billion in hospitalization costs [6]. These benefits extend beyond hospitals—primary care settings that employ dietitians have reported returns as high as NZ$99 saved for every NZ$1 invested [7].

One investment model that has been effective in enhancing care for children with cancer has been proposed by IIPAN, which provides a structural framework for the delivery of clinical nutrition care in low- and middle-income countries focused on NCDs. With the cost per nutrition consultation as low as US$1.60, this model has led to children and adolescents receiving the same standard of nutrition care available to most children in a high-income setting [33]. While IIPAN’s program offers a successful model for integrating nutrition care into hospital systems for children with NCDs, donor-supported initiatives like IIPAN’s cannot reach every child in need, underscoring the requirement for government commitment and sustainable financing to close gaps in care.

IIPAN’s model illustrates how dietitians serve as an integral part of the multidisciplinary care team, with nutrition professionals working alongside physicians, nurses, psychologists, and social workers to ensure that dietary interventions are fully integrated with medical treatment plans. Evidence from diverse clinical settings supports this approach. In type 2 diabetes care, integrating dietitians into multidisciplinary teams has been associated with greater achievement of optimal diabetes management targets [34]. In primary care, the inclusion of dietitians has improved patient-centered care, enhanced staff nutrition knowledge, and generated cost savings through medication optimization related to nutrition support [35]. Similarly, in critical illness, co-management by dietitians has been shown to shorten hospital stay and improve outcomes [36]. Collectively, these findings reinforce the vital role of dietitians within the multidisciplinary healthcare team to optimize clinical outcomes and efficiency of care.

## 3. Expanding Access to Nutrition Care

Scaling evidence-based models such as IIPAN’s requires sustained investment across multiple domains to meet the growing global demand for nutrition services. A critical barrier to this scale-up is the severe shortage of trained nutrition professionals—particularly in low- and middle-income countries. Africa and South-East Asia, for example, have just 0.9 and 1.6 nutrition professionals per 100,000 people, respectively—well below the World Health Organization’s recommended benchmark of 10 per 100,000, which is met by only 18% of countries [37]. However, this metric may not account for nutrition practitioners, such as community health workers, who play a critical role in providing nutrition care and education to families. Past attempts to integrate nutrition into health systems have relied largely on overstretched delivery platforms and inadequately trained personnel, severely limiting their effectiveness [10]. In Malawi, for instance, where there are only 11 clinical dietitians in the entire country, a government initiative introduced two dietitians in tertiary-level health facilities, yet the patient-to-dietitian ratio remained too large to meet the patients’ needs [38]. To ensure the successful and sustainable delivery of nutrition services, a significant expansion and strengthening of the nutrition workforce is needed urgently.

Expanding the number of nutrition professionals is critical; in West Africa, for instance, the number of graduates in nutrition is just one-third of what is required to meet population needs [39]. However, to ensure that increased workforce capacity translates into high-quality nutrition care, training programs and professional competencies for nutrition professionals must be strengthened. In a survey covering 18 African countries, only seven required dietitians to pass a certification exam prior to engaging in clinical practice [40]. This is similar across many lower-income countries, which lack national license and qualification systems for dietitians and nutritionists [12]. In some regions, individuals with little to no background in nutrition could be given the title of a nutritionist [41]. Establishing quality standards, through national qualifying exams, board certification, and continuing education requirements, is essential to ensure evidence-based, high-quality nutrition care.

Investment must be directed also toward comprehensive education and ongoing continuing education and training opportunities for healthcare workers. In countries with available data, fewer than half of general practitioners can accurately apply evidence-based nutrition care [42,43]. Most healthcare workers delivering nutrition services, including community health workers, receive fewer than 20 h of pre-service training in nutrition, and many educators lack the tools and expertise to deliver evidence-based nutrition care [12]. Yet the impact of improved training is clear—general practitioners who received nutrition education were 30% more likely to provide appropriate dietary advice, and 82% of trained community health workers demonstrated improved communication skills following nutrition education [44].

A well-trained workforce can only be effective if they have access to essential and culturally acceptable nutrition products. A review of integrated nutrition programs within health systems identified limited availability of nutrition products as a barrier to successful implementation, and led to reduced community trust in the program [10]. Ready-to-use therapeutic food, a standard treatment for severe acute malnutrition, can cost US$41–51 per child throughout the treatment course, limiting its sustainability and accessibility [45]. Locally produced ready-to-use therapeutic food using regional recipes with local ingredients can be as effective as commercial products and are often more acceptable to children [46]. Clinicians have used locally enriched family foods for nutrition rehabilitation successfully for over half a century [47]. IIPAN’s scalable Food as Medicine program equips hospital kitchens to prepare nutrient-dense porridges, protein-rich foods, and fortified smoothies that supplement children’s diets.

To assess the impact of the proposed initiatives comprehensively, monitoring frameworks must incorporate nutrition within the context of childhood NCD care. While there is currently no single framework that integrates nutrition monitoring specifically for children with NCDs, existing frameworks, such as the Global Nutrition Monitoring Framework, include relevant nutrition indicators like nutrition professional density and the prevalence of stunting, overweight, and wasting [48]. These existing frameworks can be adapted by adding indicators tailored to this population, such as time from NCD diagnosis to nutrition consultation, changes in nutrition status over the course of treatment, and the proportion of children and caregivers receiving nutrition counseling.

Finally, emerging technologies present powerful opportunities to bridge critical gaps in nutrition care. Telehealth offers a practical way to address workforce shortages by expanding access to specialized nutrition care, particularly for children in rural or underserved settings. Telehealth interventions have demonstrated success already in delivering nutrition counseling, leading to measurable weight loss among individuals with overweight or obesity [49]. Unfortunately, the reliance on stable internet or phone service can limit access. Artificial intelligence also holds promise for enabling more efficient, personalized nutrition care [50]. Beyond service delivery, technology can strengthen health information systems, integrating nutrition indicators and enabling more efficient data collection and analysis [12]. A joint report by the World Health Organization and the International Telecommunication Union estimated that investing just US$0.24 per person per year in digital health solutions could save over 2 million lives and prevent 7 million acute events and hospitalizations related to NCDs within the next decade [51].

## 4. Conclusions

**Figure 1:**
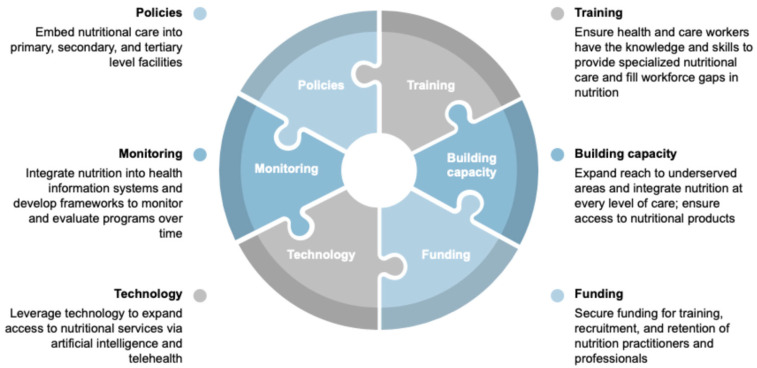
Building Sustainable Health Systems that Integrate Nutrition into Childhood Non-Communicable Disease Management.

No single resolution can solve the complex challenge of incorporating nutrition into healthcare systems. NCDs result from factors that span multiple socioecological layers, and progress will require coordinated efforts across all of them. What is needed is a bold, multisectoral response rooted in collaboration, innovation, and shared responsibility. Given these insights and the urgent need for action, we call on all partners in global health and nutrition to join us in our initiative to incorporate nutrition services into healthcare systems by focusing on six key areas (Figure 1). Policies should embed nutrition care into primary, secondary, and tertiary levels of healthcare, ensuring that nutrition care is accessible to all children. Training must equip healthcare workers with the competencies to deliver evidence-based nutrition care. Building capacity involves expanding access to nutrition services in underserved areas and ensuring the availability of culturally acceptable, affordable, and sustainable therapeutic foods and formulas. Funding should be secured through Ministries of Health to ensure sustainable health financing mechanisms that support nutrition services, thereby reducing the reliance on external funding. Technology can be leveraged to extend access to nutrition services through telehealth, digital tools, and artificial intelligence. Finally, monitoring requires the integration of nutrition-specific indicators into health information systems and the development of evaluation frameworks to track patient outcomes over time. Together, these six areas of policy, training, capacity building, financing, technology, and monitoring form the foundation for integrating accessible, high-quality nutrition care into health systems for children with NCDs. With these actions, we can ensure that every child living with an NCD has access to the highest standard of care–not only to survive, but to thrive.

## Data Availability

No new data were created or analyzed in this study. Data sharing is not applicable to this article.

## Abbreviations

The following abbreviations are used in this manuscript:

NCD	Non-communicable disease
IIPAN	International Initiative for Pediatrics and Nutrition
